# Recent advances in the understanding and therapeutic management of mastocytosis

**DOI:** 10.12688/f1000research.19463.1

**Published:** 2019-11-22

**Authors:** Julien Rossignol, Laura Polivka, Leila Maouche-Chrétien, Laurent Frenzel, Patrice Dubreuil, Olivier Hermine

**Affiliations:** 1 French Reference Center for Mastocytosis (CEREMAST), Necker Children's Hospital, APHP, Paris, France; 2 Department of Hematology, Gustave Roussy Institute, Paris-Saclay University, Villejuif, France; 3 Imagine Institute, Paris University, INSERM U 1183, Paris, France; 4 Department of Dermatology, Necker Children's Hospital, APHP, Paris, France; 5 Department of Hematology, Necker Children's Hospital, APHP, Paris, France; 6 Centre de Recherche en Cancérologie de Marseille, Inserm U1068, Marseille, France

**Keywords:** Mastocytosis, physiopathology, treatment, management, leukemia, mast cell

## Abstract

Mastocytosis is a rare disease due to the abnormal accumulation of mast cells in various tissues. Its clinical presentation is heterogeneous depending on mast cell infiltration and mediators release. In some cases, it is associated with hematological malignancies. Prognosis varies from very good with a life expectancy similar to the general population in indolent forms of the disease to a survival time of just a few months in mast cell leukemia. Although in most cases a somatic *KIT *D816V mutation is found in tumor mast cells, the physiopathology of the disease is not yet fully understood. Additional germline and somatic mutations may explain this heterogeneity. Treatments aim at blocking effect of mast cell mediators, reducing mast cell activation and tumor burden. New drugs mainly directed against the tyrosine kinase activity of KIT have dramatically changed the quality of life and prognosis of mast cell diseases. Present and future therapeutic strategies are discussed in this review.

## Introduction

Mastocytosis covers a spectrum of disease characterized by the clonal expansion and accumulation of mast cells (MCs) in the skin and in various internal organs, including the bone marrow (BM), spleen, lymph nodes, and gastrointestinal tract ^1, 2^. The disease presentation is heterogeneous and ranges from cutaneous mastocytosis (CM), which often resolves spontaneously in children, to acute MC leukemia (MCL), with a survival time of just a few months.

In 2016, the World Health Organization (WHO) classified types of mastocytosis into three main groups: CM, with involvement limited to the skin, systemic mastocytosis (SM), with the additional infiltration of tissues other than the skin, and MC sarcoma ^3^. SM is then subdivided into indolent SM (ISM) and, if organ infiltration leads to organ dysfunction, advanced SM (AdvSM). CM and ISM are by far the most frequent subtypes. Although patients with CM or ISM have a normal life expectancy, their quality of life may be impaired by symptoms related to MC activation ^4^. In contrast, AdvSM is associated with a decrease in life expectancy in the vast majority of patients; this is because of abnormal infiltration of MCs into various organs and thus impairment of the latter’s physiological functions ( Table 1).

**Table 1.** The 2016 revision of the World Health Organization classification of mastocytosis ^3^.

**• Cutaneous mastocytosis** Urticaria pigmentosa, maculopapular cutaneous mastocytosis, diffuse cutaneous mastocytosis, mastocytoma of skin • **Systemic mastocytosis** a. Indolent systemic mastocytosis (ISM) * b. Smoldering systemic mastocytosis (SSM) * c. Systemic mastocytosis with an associated hematological neoplasm (SM-AHN) † d. Aggressive systemic mastocytosis (ASM) * e. Mast cell leukemia (MCL) • **Mast cell sarcoma (MCS)**

*For a full diagnosis of these subtypes, information on B and C findings is required but may not be available at the time of the initial tissue diagnosis. †This category is equivalent to the previously described “systemic mastocytosis with an associated clonal hematologic non-mast cell lineage disease”. AHNMD and AHN can be used synonymously.

Mastocytosis is strongly linked to somatic mutations in the *KIT* gene (and especially the *KIT* D816V mutation) coding for the tyrosine kinase receptor KIT, which lead to constitutive activation ^5^. However, it is now accepted that the *KIT* D816V mutation does not fully explain the spectrum of mastocytosis diseases. Recent studies of AdvSM have shown that other somatic genetic determinants (typically involving epigenetic regulators) can explain the disease’s heterogeneity. Most of these variants are associated with myeloid neoplasms and may worsen the prognosis ^6, 7^. In other cases in which *KIT* mutations are not found, the disease’s physiopathology has yet to be characterized.

The therapeutic management of mastocytosis is still a challenge for the physician ^8^. For patients with ISM, the treatment objective is to decrease the severity of MC activation and thus improve quality of life. Nevertheless, the treatment must be well tolerated and, if possible, devoid of short- and long-term side effects. In AdvSM, the main treatment goal is to prolong survival in patients with comorbidities and poor general status.

Here, we will review new insights into the diagnosis, pathogenesis, and therapeutic management of mastocytosis and cover perspectives for research into this complex disease.

## Diagnosis

Mastocytosis can potentially affect all of the organs and can cause a wide variety of clinical manifestations ^1^. The clinical signs are categorized into two types: those directly related to MC infiltration (skin, spleen, bone, etc.) and those related to MC activation.

Since skin involvement is easy to see, most cases of mastocytosis are revealed by the cutaneous manifestations like flushes, pruritus, and specific lesions. Alternatively, mastocytosis may be diagnosed after patients present with various non-specific symptoms that mimic conditions such as irritable/inflammatory bowel disease, chronic fatigue syndrome, fibromyalgia, and osteoarthritis. Constitutional symptoms (such as fatigue, pain, and neurologic and psychiatric symptoms) are also frequent but may not prompt a diagnosis of mastocytosis in the absence of skin involvement. Recurrent, severe, idiopathic anaphylaxis is more frequent in patients with mastocytosis and is especially associated with Hymenoptera stings, food allergy, exercise, and adverse drug reactions. The REMA score that includes gender, tryptase level, and clinical signs like syncope might be a useful decision-support tool, i.e. for deciding whether or not to perform the full diagnostic work-up for mastocytosis in patients with anaphylaxis, particularly in idiopathic cases or following Hymenoptera stings ^9, 10^. Recently, Carter *et al*. have reported the “NIH Idiopathic Clonal Anaphylaxis Score” using the aforementioned criteria with allele-specific quantitative PCR for the *KIT* D816V mutation. Interestingly, this score has better positive and negative predictive value than the REMA score ^11^.

Early onset osteoporosis is a classical manifestation of mastocytosis and typically involves trabecular bones. Less frequently, the disease is diagnosed during investigations of an associated hematologic neoplasm, such as a myeloproliferative disorder, myelodysplastic disease, or acute myeloid leukemia.

Lesions associated with MC infiltration are secondary to increased proliferation and survival of pathological MCs. On the other hand, the signs related to MC activation are secondary to the release of intracellular mediators after activation of the MC. Schematically, three phases succeed each other during this activation ^12^. First, MC degranulation of prestored mediators occurs a few seconds after triggering activation. These mediators include histamine, tryptase, proteoglycan, and cytokines. This first step is followed by the second phase characterized by the release of neo-synthesized mediators such as prostaglandin (PGD2), leukotrienes, and platelet-activating factor. Early mediators (histamine, PGD2, and leukotrienes) contribute to most of the signs of MC activation (pruritus, urticaria, flushing, hypotension, anaphylactic shock, edema, abdominal pain, and diarrhea). Finally, MCs secrete pro-inflammatory cytokines (TNFα, IL-1, and IL-6), pro-TH2 cytokines (IL-5 and IL-13), and other cytokines (TGFβ, VEGF, and FGF) that may participate in tissue lesions.

## Classification

Owing to the heterogeneity of the disease, and in order to better evaluate the prognosis and define treatment goals and endpoints, the WHO defined mastocytosis as a specific entity. Hence, because of its unique clinical and pathologic features, mastocytosis is no longer considered a subgroup of myeloproliferative neoplasms. Mastocytosis is classified into three main groups: CM (i.e. involving only the skin), SM (involving organs other than the skin), and MC sarcoma ^3^. Five major variants of SM have been defined: ISM, smoldering SM (SSM), SM with an associated hematologic neoplasm (SM-AHN), aggressive SM (ASM), and MCL ( Table 1).

CM requires a histologic confirmation of MC infiltration into the skin and the absence of any other organ involvement (and especially not the BM) ^13^. Cutaneous lesions are then divided into maculopapular CM (MPCM, also known as urticaria pigmentosa), diffuse CM, and localized mastocytoma of the skin. Telangiectasia macularis eruptiva perstans is a controversial clinical entity and was not recognized by the WHO. Although CM lesions are present in CM and SM at all ages, they are especially visible in all pediatric cases and less frequent in older adults. Therefore, CM is by far the most common form of mastocytosis in childhood, whereas AdvSM is extremely rare. However, the frequency of isolated CM is difficult to evaluate because BM aspirates and biopsies are rarely collected in children. Mastocytoma (a hemispheric, firm nodule) is the most common cutaneous manifestation of mastocytosis observed before the age of 3 months. Two distinct forms of MPCM have been recognized in children: a variant characterized by monomorphic, small lesions (formerly called urticaria pigmentosa) and one characterized by polymorphic (often large) lesions; these are now referred to as MPCM-small lesions and MPCM-large lesions, respectively ^14^. Diffuse CM is a rare variant that generally presents during the neonatal period. It is characterized by generalized erythema and is usually associated with pachydermia. In adult-onset mastocytosis, MPCM-small lesions are by far the most common cutaneous manifestations; other forms are very rare.

When the mastocytosis develops in adulthood, it is usually systemic. However, it is likely that some of these cases will be diagnosed as CM if high-sensitivity techniques are not used to detect systemic MC involvement. Nevertheless, in a trial of masitinib’s efficacy, the clinical response was better in cases with documented systemic involvement, suggesting that CM and SM are indeed distinct entities ^15^. A diagnosis of SM requires the documentation of mastocytosis away from the skin. The BM and the gastrointestinal tract are frequently involved, although MC infiltration can be found in other organ systems (bones, the liver, etc.).

**Figure 1.**
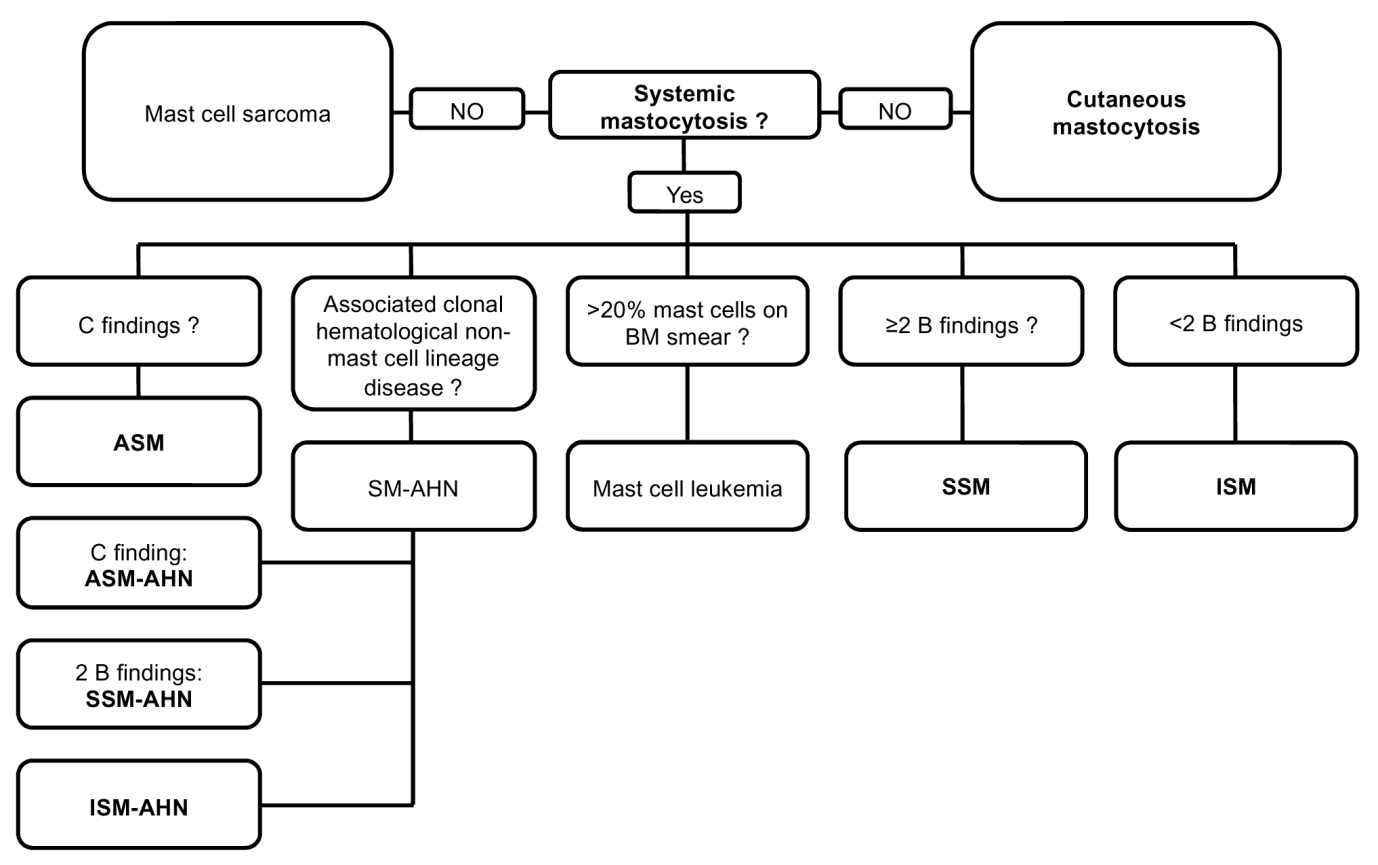
Diagnostic flow chart for mastocytosis. AHN, associated hematologic neoplasm; ASM-AHN, aggressive systemic mastocytosis with an associated hematologic neoplasm; BM, bone marrow; ISM, indolent systemic mastocytosis; MCL, mast cell leukemia; SM-AHN, systemic mastocytosis with an associated hematologic neoplasm; SSM, smoldering systemic mastocytosis; SSM-AHN, smoldering systemic mastocytosis with an associated hematologic neoplasm.

To further characterize the disease, its severity must be characterized ( Figure 1) ^3^. The disease features are referred to as “B findings” if a high MC burden and an expansion of the neoplastic process into multiple hematopoietic lineages are present, with no obvious impairments in organ function, and “C findings” if the MC infiltration has produced organ dysfunction ( Table 2) ^3, 16^. Both ISM and ASM can be associated with a hematologic neoplasm (i.e. SM-AHN). Most hematologic malignancies associated with mastocytosis are of myeloid origin (myelodysplastic syndrome, acute myeloid leukemia, and myeloproliferative neoplasm). In order to define the best therapeutic strategy in cases of SM-AHN, it is essential to assess whether C findings are due to the SM or the AHN.

**Table 2.** B and C findings for the diagnosis of systemic mastocytosis.

B-findings	C-findings
(1) High MC burden : Infiltration grade (MC) in BM > 30% in histology and serum tryptase 200 ng/ml (2) Dysmyelopoiesis with normal blood counts or slight persisting deviation without progression (3) Organomegaly ( **without impaired** **organ function**) : Palpaple hepatomegaly or/and lymphadenopathy palpable or visceral lymph node-enlargement found in US or CT (>2cm) or/and palpable splenomegaly.	Organopathy ( **with impaired organ function**) **:** (1) Bone marrow: cytopenia(s) ANC < 1000/µl Hemoglobin < 10g/dl Platelet < 100 000/µl (2) Liver: palpable hepatomegaly with ascites, abnormal liver function tests and/or portal hypertension (3) Spleen: palpable splenomegaly with hypersplenism (4) GI tract: malabsorption with hypoalbuminemia and weight loss (5) Skeleton: bone lesions with large osteolyses or/and severe osteoporosis with pathologic fractures

ANC, absolute neutrophil count; BM, bone marrow; CT, computed tomography; GI, gastrointestinal; Hb, hemoglobin; LN, lymph node; MC, mast cell; Plt, platelet; US, ultrasound. Adapted from Valent *et al*. ^16^

MCL is characterized by the presence of an abnormally high proportion of MCs (>20%) on BM aspirate smears. Although MCL is very rare, cases can be subclassified as acute MCL with C findings. Some patients with MCL do not exhibit C findings and present a protracted course. Although not included in the WHO classification, we may describe these patients as having chronic MCL with no C findings. Expression of the cell proliferation marker KI67 may distinguish between these two entities (personal communications).

At the French Reference Center for Mastocytosis (CEREMAST, Paris, France), the work-up for suspected mastocytosis includes diagnostic tests for mastocytosis, screening for B and C findings and osteoporosis, and an extensive evaluation of the functional impairments associated with MC activation. We always perform a BM aspirate and biopsy and investigate exon 17 *KIT* mutations in BM cells and the atypical CD2 and CD25 expression on MCs. If AdvSM is suspected (i.e. with the presence of C findings), we perform next-generation sequencing on BM cells and screen for any additional mutations that are characteristic of myelodysplastic or myeloproliferative disorders and have prognostic and therapeutic value. In routine clinical practice, any further investigations (imaging or skin, intestinal tract, or liver biopsies, etc.) are usually prompted by abnormal clinical and laboratory features. In children, we perform only a BM aspirate and biopsy when AdvSM is suspected and the tryptase level is abnormally elevated (>200 ng/ml). In view of the high frequency of mutations in *KIT* exons 8 to 13 and 17, this gene is always sequenced in suspected cases of childhood-onset mastocytosis.

## Pathogenesis

### New insights into mast cell ontogeny

Recently, Gentek *et al*. reported that, like macrophages, MCs have two hematopoietic origins in the mouse ^17^. Unlike MCs in other organs, MCs in the embryonic skin are derived from the yolk sac. These MCs persist after birth before being gradually replaced by the definitive adult MCs. Yolk-sac-derived MCs differ phenotypically from their BM-derived counterparts and have a distinct gene expression pattern. These newly discovered differences might explain the complete, spontaneous remission observed in more than 70% of cases of childhood-onset mastocytosis.

### KIT mutations do not account for all mastocytosis phenotypes

More than 85% of patients with adult-onset mastocytosis present a somatic gain-of-function mutation in the *KIT* gene coding for the KIT transmembrane tyrosine kinase receptor ^18^. The most frequently detected mutation leads to the substitution of an aspartic acid by a valine (D816V) in the second catalytic domain, resulting in constitutive kinase activity. In turn, this promotes MC activation, survival, and proliferation independently of KIT’s ligand (stem cell factor [SCF]). Under physiological conditions, SCF is critical for MC proliferation, differentiation, survival, and migration. Stimulation (by IgE or otherwise) enhances MC activation through the Ras-MAP kinase, SRC, STAT, and PI3K/AKT pathways. A somatic *KIT* mutation is present in around 80% of cases of childhood-onset mastocytosis. However, in contrast to adult cases, only 42% of the children present a mutation in codon 816 (exon 17); 44% of cases have a mutation outside exon 17 (mostly in the fifth Ig loop of the KIT extracellular domain, encoded by exons 8 and 9) ^19^. Although all of these *KIT* mutations cause the constitutive activation of kinase activity, they cannot fully explain the clonal expansion of MCs and the heterogeneous symptoms observed in different types of mastocytosis. Patients harboring the same mutation can present with very different clinical pictures. In childhood-onset mastocytosis, complete spontaneous remission is typically observed at puberty, despite the presence of *KIT*-activating mutations, including the D816V mutation. Transgenic mice overexpressing *KIT* D816V exhibit slow expansion and indolent accumulation of MCs in the tissues, with incomplete penetrance, and they rarely develop aggressive mastocytosis ^20^. Although most patients with mastocytosis did not have a family history of MC disease, around 100 familial cases worldwide have been reported in the literature. However, only 13 of these presented with germline KIT mutations (A533D, M541L, Del419D, S451C, K509I, V559A, R634W, or N822I) ^21– 31^. In the mastocytosis cohort at the CEREMAST, familial forms appear to be more prevalent than in the literature. Indeed, about 7% of our patients have a first- or second-degree relative with mastocytosis. The majority of these relatives do not have a mutation in the *KIT* gene (unpublished data). Thus, *KIT* mutations do not account for all familial forms of the disease. Lastly, BaF3 cells overexpressing *KIT* D816V do not display a transformed phenotype and are even driven towards maturation by the mutation ^32^.

Taken as a whole, these observations strongly suggest the additional involvement of one or more germline or somatic mutations or polymorphisms in genes other than KIT; these would act synergistically with *KIT* mutations in the development of mastocytosis.

### Associated somatic mutations

Somatic mutations in other genes (notably epigenetic regulator genes, such as *SRSF2, ASXL1, RUNX1*, and *TET2*) have been described in ASM and are often found in patients with a myeloid neoplasm. These mutations account for, at least to some extent, the different clinical entities in mastocytosis.

**Figure 2.**
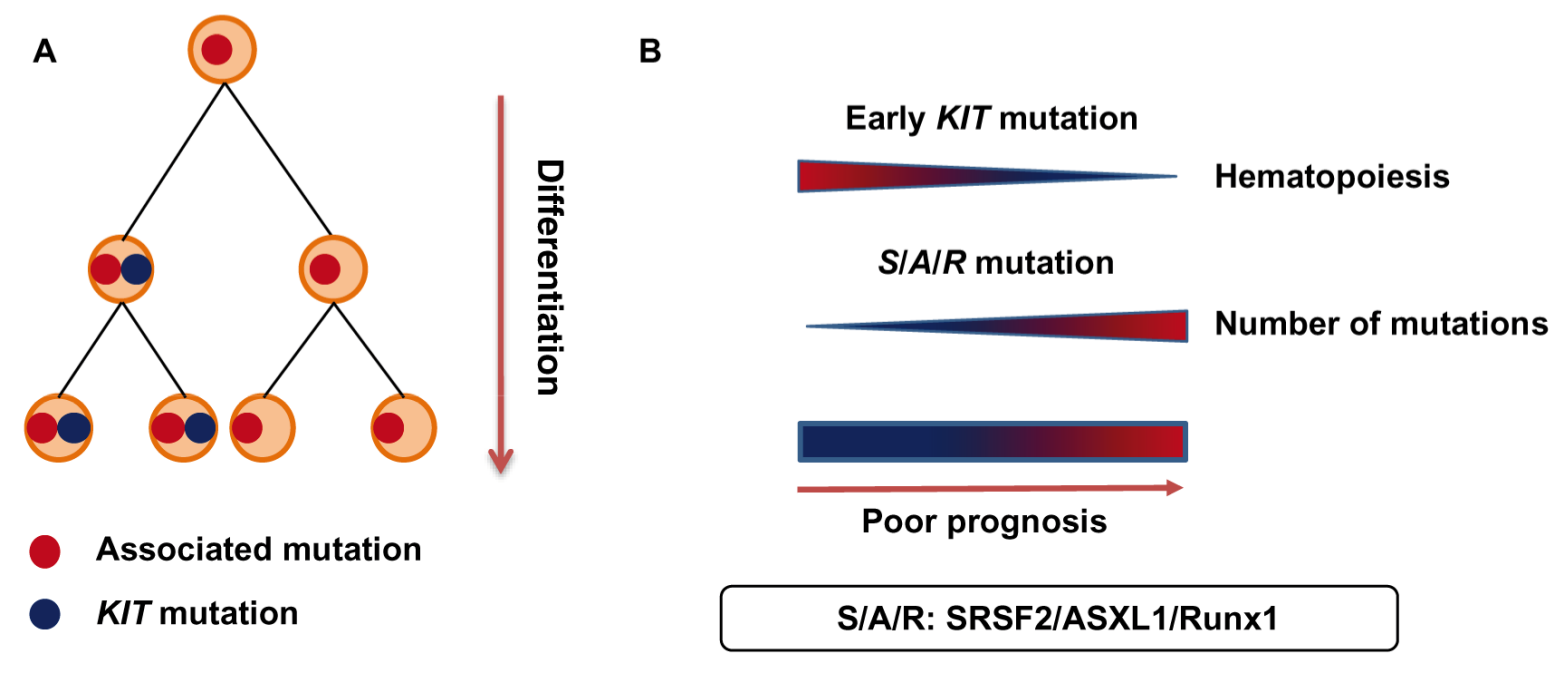
Advanced systemic mastocytosis: clonal architecture and prognosis. **A**. A representation of the clonal architecture of the hematopoietic compartment in advanced mastocytosis. The associated mutations occur before the *KIT* mutation. **B**. An early D816V *KIT* mutation inside the hematopoietic progenitor compartment and the presence of *SRSF2*, *ASXL1*, *RUNX1* (S/A/R) mutations are associated with a poor outcome. A, *ASXL1* mutation; R, *RUNX1* mutation; S, *SRSF2* mutation.

Several studies have investigated the prognostic impact of these additional mutations. Most of the mutations have been found in patients with AdvSM, and the presence and number of *SRSF2, ASXL1*, and *RUNX1* (S/A/R) mutations are associated with a poor prognosis, even in patients treated with midostaurin ( Figure 2) ^33– 35^. *In vitro* studies suggest that these mutations act in synergy with abnormal KIT signal transduction to induce MC survival and proliferation. However, the underlying molecular mechanisms have yet to be characterized.

Two studies have investigated the clonal architecture in SM-AHN. First, Schwaab and colleagues reported a correlation among the *KIT* allele burden, the number of colony-forming unit granulocytes monocytes (CFU-GM) bearing the D816V *KIT* mutation, and the severity of mastocytosis. This finding strongly suggests that an early D816V *KIT* mutation inside the hematopoietic progenitor compartment is associated with a poor outcome ( Figure 2) ^6^. The second study of mutations in both AHN and mastocytosis showed that the D816V *KIT* mutation could be found in CD15 ^+^ circulating cells and that MCs carried associated mutations. The results of a colony forming unit assay suggested that the associated mutations arise before the *KIT* mutation during hematopoiesis ^33^.

## Prognosis

The prognosis for CM and ISM is excellent, as reported by Lim *et al*. in 2009 ^36^. Indeed, patients with ISM in the USA have much the same life expectancy as the general population. However, the prognosis is poor for AdvSM, especially in elderly patients: median OS was 41 months for ASM, 24 months for SM-AHN, and 2 months for MCL in the aforementioned study reported by Lim *et al*.

Along with age, several prognostic factors have been reported. Splenomegaly and elevated alkaline phosphatase are predictive of a poor prognosis in both AdvSM and ISM. In AdvSM, the presence of one or more S/A/R mutations has a negative impact on the outcome.

## Treatment

Several new treatments have improved the prognosis and quality of life for patients with mastocytosis. The therapeutic objective depends on the type of mastocytosis, the prognosis, the patient’s age, and the presence or absence of an associated hematologic neoplasm.

### Cutaneous and indolent systemic mastocytosis: the main treatment objective is to decrease handicap and improve quality of life ( Table 3 and Figure 3)

**Figure 3.**
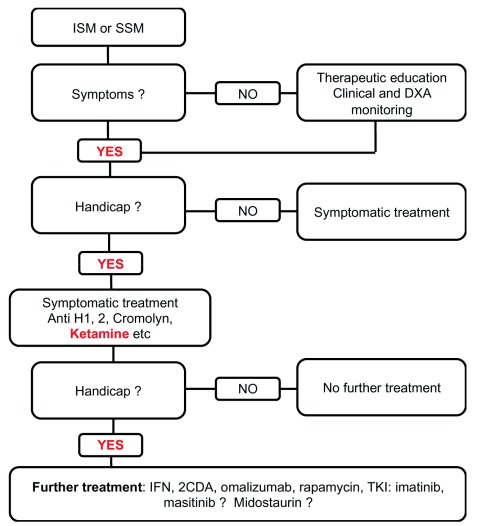
Treatment flow chart for indolent and smoldering systemic mastocytosis. 2CDA, cladribine; DXA, dual-energy X-ray absorptiometry; IFN, interferon; ISM, indolent systemic mastocytosis; SSM, smoldering mastocytosis; TKI, tyrosine kinase inhibitor.

**Figure 4.**
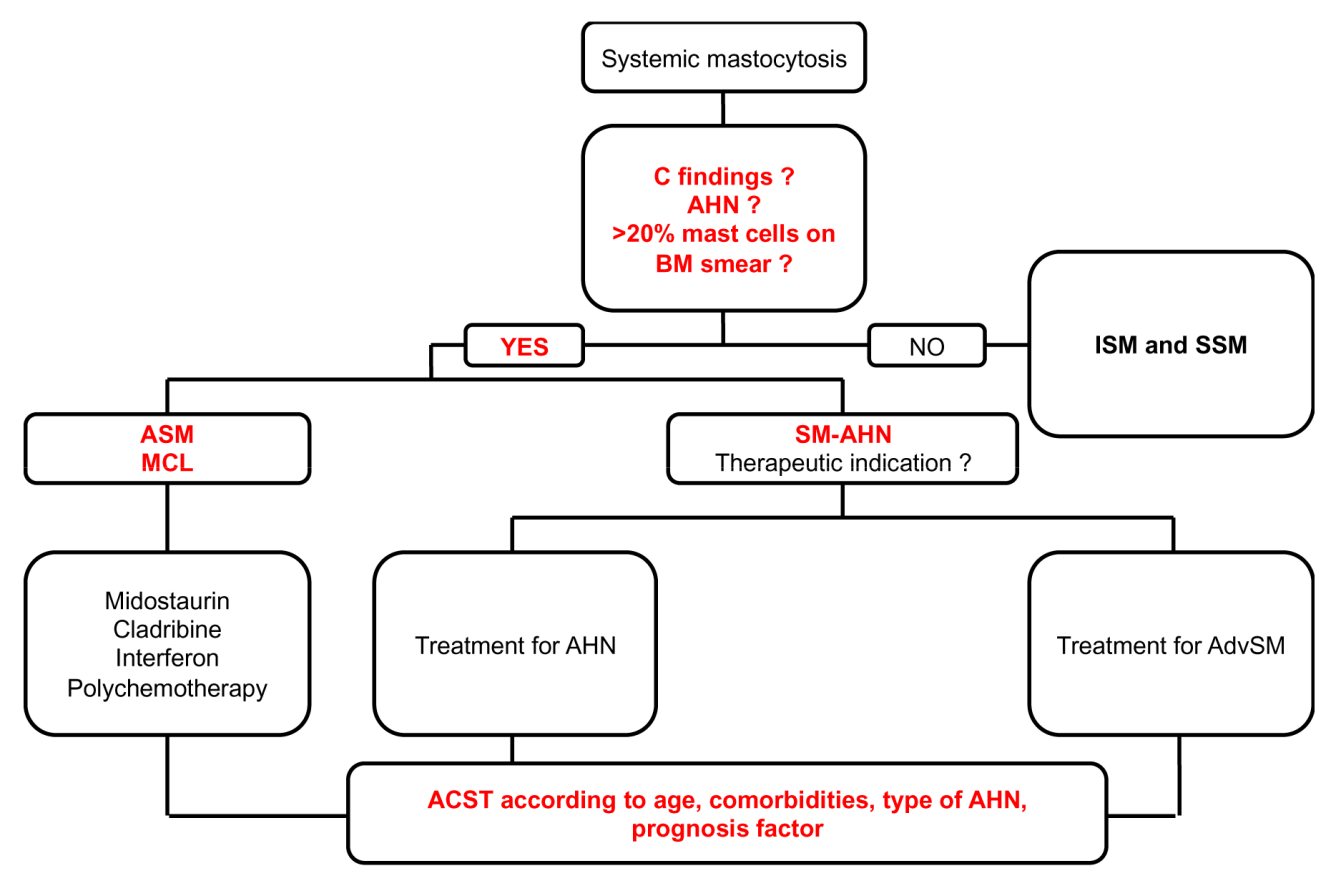
Treatment flow chart for advanced systemic mastocytosis. ASCT, allogeneic stem cell transplantation; AdvSM, advanced systemic mastocytosis; AHN, associated hematologic neoplasm; ASM, aggressive systemic mastocytosis; BM, bone marrow; ISM, indolent systemic mastocytosis; MCL, mast cell leukemia; SM-AHN, systemic mastocytosis with an associated hematologic neoplasm; SSM, smoldering systemic mastocytosis.

Patients with an indolent form of mastocytosis (like CM and ISM) can present two types of clinical symptoms: those related to MC activation syndrome (MCAS) and those related to MC infiltration (particularly skin infiltration). The treatment choice is therefore based on the signs that cause the most severe disability ( Figure 3 and Figure 4).

**Table 3.** Completed clinical trials and large retrospective studies of indolent systemic mastocytosis or smoldering systemic mastocytosis.

Author (Journal)	Treatment	Patients	Type of study	ORR (%)	Follow up
Barete (Blood) ^40^	Cladribine	36	Retrospective	ORR: 92 %	Duration of response: 44.5 months
Lim (Am. J. Hematol.) ^41^	Interferon	11	Retrospective	ORR: 60%	Duration of response: 12 months (with AdvSM)
Broesby-Olsen (Allergy) ^42^	Omalizumab	13	Retrospective	ORR: 78.6 %	Duration of treatment: 21 months
Lemal (JACI practice) ^43^	Omalizumab	56	Retrospective	ORR: 76.8%	Response at least > 3 months (76.7%)
Vega-Ruiz (Leukemia Research) ^44^	Imatinib	11	Phase II	ORR: 55%	Duration of improvement lasted between 9 and 36 months
Verstovsek (Clin Cancer Res) ^45^	Dasatinib	18	Phase II	ORR: 33%	Duration of symptomatic responses ranged from 3 to 18+ months
Lortholary (Lancet) ^15^	Masitinib	masitinib n=71 placebo n=64	Phase III	Cumulative response (≥75% symptom improvement) Masitinib: 18·7% Placebo: 7.4%	Duration of treatment: 18·9 months
Moraly (unpublished data, submitted for publication)	Rapamycine	11	Retrospective	ORR: 70%	Duration of treatment: 10.8 months

AdvSM, advanced systemic mastocytosis; ORR, overall response rate.

The treatment of signs of MC activation is initially based on antihistamines (anti-H1 and anti-H2), leukotriene receptor inhibitors, and cromolyn. Although aspirin and NSAIDs may induce allergic and/or anaphylactic reactions, there is no absolute contra-indication to these drugs and they may even improve some symptoms like pains and flushing. For children with isolated mastocytoma and severe symptoms, the treatment of choice is topical corticosteroids with occlusive dressings. If this fails, excision *in toto* can be considered. Ultraviolet A (either alone or combined with oral psoralen) can be used in adolescents and adults to treat skin manifestations (especially pruritus) that are resistant to standard medications. It is essential to eliminate triggering factors (e.g. foods, medications, or environmental factors such as Hymenoptera stings) by testing for IgE-mediated allergy. These measures must be accompanied by patient education on epinephrine self-administration (especially in patients with a history of anaphylactic reactions) ^8^. Honeybee- and wasp-allergic patients with a history of severe reactions must be submitted to allergen-specific immunotherapy ^37^. Patients must be warned of the risk associated with anesthesia; for example, the French Society of Anesthesia & Intensive Care Medicine has published specific guidelines on this topic. Most patients are improved by these initial therapeutic measures ^38^.

In our experience, we prescribe anti-H1 medications almost systematically for MCAS, especially for pruritus, urticaria, flushing, recurrent hypotension/anaphylaxis, or neuropsychic manifestations. We use anti-H2 medication and cromolyn with or without a proton pump inhibitor for gastrointestinal symptoms. Leukotriene antagonists seem particularly effective for flushing and pollakiuria. We are currently testing the effect of NMDA receptor inhibitors (such as ketamine) in patients (about 20%) with fentanyl-refractory chronic pain ^39^. Our preliminary results suggest that these treatments can be efficient. Lastly, as there is no prospective study published so far on the role of bone-forming therapies for mastocytosis-associated osteoporosis, we are investigating the efficacy of denosumab in a phase III study (NCT03401060). In addition to signs of MC activation, patients may experience disability due to the psychological and social impact of the skin involvement in MPCM. It is difficult to accurately assess the disability related to acute and chronic symptoms. Several consultations are necessary to confirm the patient's willingness to accept the potential risks associated with certain treatments, particularly for esthetic reasons, because the treatment of cutaneous lesions requires the use of cytoreductive drugs.

At present, there are no guidelines on MC activation that is refractory to antihistamine medications or on when cytoreductive drugs should be used. With the exception of masitinib, prospective clinical trials have not been performed. Hence, treatment choice is based solely on the specialist center’s own experience ( Table 3) ^15, 40– 45^.

***Tyrosine kinase inhibitors.*** Given the involvement of constitutive KIT tyrosine kinase activity in the pathophysiology of mastocytosis, several drugs used to block both BCR-ABL and KIT in cases of chronic myeloid leukemia have also been evaluated in SM.

The results of several prospective studies of first-generation (imatinib) and second-generation (dasatinib and nilotinib) inhibitors have been disappointing, with low response rates ^44, 45^. On the molecular level, the presence of the D816V *KIT* mutation in the vast majority of cases of mastocytosis confers resistance to the above-mentioned inhibitors. We therefore reserve these treatments for the small proportion of patients who do not have a *KIT* exon 17 mutation; the results for MC activation and cutaneous involvement are encouraging.

Masitinib was evaluated in the first phase III study of patients with CM or ISM and significant disability ^15^. Although masitinib lacks direct activity on the D816V *KIT* mutation, its action (particular on Lyn) appears to block MC degranulation *in vitro*. In Lortholary *et al*.’s study, masitinib had an acceptable safety profile and showed significant efficacy on symptoms; a reduction of more than 75% in symptoms was observed in 18.7% of the patients treated with the inhibitor and 7.4% of those treated with placebo ( *P* = 0.0076). This drug is currently being evaluated in a confirmatory phase III trial.

***Cladribine.*** Cladribine has never been prospectively evaluated in SM, although long-term follow-up data from patients with SM have been reported retrospectively. This drug has a positive effect on both skin involvement and the symptoms of MC activation. Barete *et al*. studied cladribine treatment in 36 patients with ISM/SSM; the overall response rate was 92%, and the median response duration was 44.5 months ^40^. Although the treatment induced significant neutropenia and prolonged CD4 ^+^ T-cell lymphopenia, the toxicity was mild and acceptable for an indolent disease. However, given the risk of secondary cancer observed in other indications, it is recommended to exclude patients with a history of cancer and to limit the number of courses of cladribine (0.14 mg/kg/day, for 5 days) to 6. We are currently looking at whether lower-dose maintenance treatment with cladribine might be as efficacious.

***Omalizumab.*** Omalizumab is an anti-IgE monoclonal antibody indicated in the treatment of asthma and chronic urticaria. Given the drug’s mode of action and lack of cytoreductive activity, it has been suggested that omalizumab can decrease the signs of MC activation associated with mastocytosis ^42^. The initial results in a small cohort were encouraging. In an analysis of the CEREMAST database, we reported on the largest-yet cohort of omalizumab-treated patients (n = 56) with indolent, cutaneous forms and MCAS ^43^. The results were particularly encouraging, with an overall response rate of 76.8% and reductions in all symptoms assessed, even the difficult-to-treat neuropsychiatric symptoms. Omalizumab is therefore an option that we often consider in patients who are very disabled by their signs of MC activation. Further work is needed to define the population that will benefit most from omalizumab treatment. At present, the optimal dosing frequency (150 to 300 mg every week to every four weeks) remains to be determined. Furthermore, omalizumab may be of value for Hymenoptera venom desensitization in patients with mastocytosis.

***Other options.*** Pegylated interferon (pegIFN) has been used for decades in the treatment of mastocytosis. It effectively reduces both the signs of MC activation and the extent of cutaneous lesions ^41^. The main limitation relates to the associated neuropsychiatric side effects in a patient population that frequently suffers from psychiatric signs in general and major depressive episodes in particular. However, pegIFN remains a valuable option, especially when other treatments are contraindicated or for cladribine sparing. PegIFN can be used also to treat the myeloproliferative disorders associated with mastocytosis.

Rapamycin may also be a relevant treatment option in relapsed or refractory patients carrying the D816V *KIT* mutation. We found that *in vitro*, D816V-mutated MC lines (but not wild-type MC lines) were specifically sensitive to rapamycin ^46^. Even though the first-in-human results did not show significant efficacy, our study of a series of 24 patients (including 12 with ISM) highlighted a response rate of 70% (especially for the signs of MC activation), as could be expected from the *in vitro* data (unpublished data, submitted for publication). Efficacy on MPCM was also observed.

### Advanced mastocytosis: the main objective is to prolong overall survival ( Table 4 and Figure 4)

Advanced mastocytosis is a spectrum of hematological malignancies with a poor prognosis. In eligible and particularly poor prognosis patients, the aim of the treatment is to induce remission to perform an allogeneic stem cell transplantation (ASCT) that remains the only current treatment with the potential to cure. Until recently, no targeted therapies were available for this difficult-to-treat set of MC diseases ( Table 4) ^40, 41, 44, 45, 47– 50^. Lately, impressive efficacy of midostaurin has been reported in the first large-scale phase II study in advanced mastocytosis, and so it was approved by the FDA in 2017. We will discuss below the main cytoreductive therapies used at our reference center.

**Table 4.** Completed clinical trials and large retrospective studies for advanced mastocytosis.

Author (Journal)	Treatment	Patients	Type of study	Non hematological AE (grade III/IV)	Response	Mediane response duration	Overall survival
Lim ( *Am J* *Hematol*.) ^41^	Interferon α	30	Retrospective	Asthenia, depression	ORR: 50%	12 months (with ISM)	NA
Kluin-Nelemens ( *Blood*) ^47^	Cladribine	6	Phase II	Infection, toxidermia	ORR: 100% CR: 0%	NA	NA
Barete ( *Blood*) ^40^	Cladribine	32	Retrospective	Infection	ORR: 50% CR: 0%	ASM: 30 months SM-AHN: 57 months	Median: ASM: 2.4 years SM-AHN: 6.5 years
Vega-Ruiz ( *Leukemia* *Research*) ^44^	Imatinib	9	Phase II	Ascites, diarrhea, asthenia, pain, rash	ORR: 11% CR: 11%	NA	NA
Lim ( *Am J* *Hematol*.) ^41^	Imatinib	19	Retrospective	Diarrhea, edema	ORR: 20% CR: 0%	19 months (with ISM)	NA
Pagano ( *Int J* *Hematol.*) ^48^	Imatinib	17	Retrospective	NA	ORR: 29% CR: 5%	NA	NA
Verstovsek ( *Clin* *Cancer Res*) ^45^	Dasatinib	15	Phase II	Pleural effusion, pains, nausea, asthenia	ORR: 13% CR: 13%	12 months	Median: 13 months
Gotlib ( *NEJM*) ^49^	Midostaurin	116	Phase II	Nausea, diarrhea, fatigue	ORR: 60% CR: 0%	24.1 months	Median: 33.9 months
Ustun ( *JCO*) ^50^	ASCT: MAC: 63% Geno-identical: 60%	57	Retrospective	1 year NRM: 20%	ORR: 70% CR: 28%	20 months	3 years overall survival: 57%

AdvSM, advanced systemic mastocytosis; AE, adverse events; ASCT, allogeneic stem cell transplantation; ASM, aggressive systemic mastocytosis; CR, complete response; ISM, indolent systemic mastocytosis; MAC, myeloablative conditioning; NA, not available; NRM, non-relapse mortality; ORR, overall response rate; SM-AHN, systemic mastocytosis with an associated hematologic neoplasm.

***Midostaurin.*** The broad-spectrum tyrosine kinase inhibitor midostaurin has activity against D816V *KIT* mutations. The results of the first large-scale clinical trial in patients with AdvSM (n = 116) were published in 2016 ^49^. Overall, 60% of the patients treated with midostaurin exhibited a response. The safety profile was acceptable, and the most frequent adverse events affected the gastrointestinal tract (nausea and diarrhea). The median response duration was 24.1 months, and the median overall survival time was 33.9 months. Interestingly, the midostaurin response rates were similar in all of the subgroups of patients with ASM, including those with AHN. However, histologically complete responses were rarely observed, and treatment had to be maintained. In our opinion, midostaurin is currently the first-line treatment of choice for first-line AdvSM.

***Cladribine.*** Cladribine has been studied to some extent, but it is not considered as the standard of care ^47^. In the above-mentioned study by Barete *et al*., 32 patients received cladribine for AdvSM ^40^. The overall response rate was 50%, and the median response durations in ASM and SM-AHN were 30 and 57 months, respectively. However, the median overall survival time was only 2.4 years for ASM. Furthermore, the treatment was not efficient in SM with AHN (particularly associated with myelodysplastic syndrome) and worsened cytopenia. Therefore, cladribine might best be used to treat smoldering forms, chronic MCL and forms associated with myeloproliferative neoplasms, or as a bridge to ASCT transplantation for midostaurin refractory patients.

***Allogeneic stem cell transplantation.*** ASCT remains the only curative treatment for AdvSM and must be considered for all patients. The procedure’s major limitation is eligibility, given the frequency of comorbidities and advanced age in this patient population. Ustun *et al*. have reported on the largest-yet published series of patients treated with ASCT for AdvSM (n = 57) ^50^. Around two-thirds of the patients underwent prior myeloablative conditioning, and the 1-year non-relapse mortality rate was 20%. Strikingly, the overall response rate was 70%, and the complete response rate was 28%. The SM and AHN components were both sensitive to ASCT. The 3-year overall survival rate was 57%. It is noteworthy that most of these patients had not received midostaurin, and so one would expect to see better results in midostaurin-treated individuals. A Europe-wide retrospective analysis is ongoing (the European Society for Blood and Marrow Transplantation registry).

At the CEREMAST, we always consider early ASCT for eligible patients with MCL or high-risk AHN ( Figure 4). For other cases, we typically use next-generation sequencing to screen for S/A/R mutations and decide on the optimal time for ASCT. With regard to the poor outcome for patients bearing these mutations, we plan to perform ASCT within a year of diagnosis (i.e. as soon as patients have achieved the best possible response to treatments such as midostaurin).

## Future perspectives

Therapeutic progress over the last 10 years (particularly with the emergence of KIT D816V kinase inhibitors) has improved quality of life and survival in patients with mastocytosis. Indeed, these patients have benefited from targeted therapies developed in other diseases, such as cancer and chronic urticaria. Nevertheless, most patients with SM relapse or are incompletely relieved (even after midostaurin treatment), and the symptoms of mastocytosis often decrease their quality of life and significantly affect their social and working lives. Hence, novel therapeutic approaches (alone or in combination with other treatments) are therefore still required, especially for patients with AdvSM who are not eligible for ASCT.

**Figure 5.**
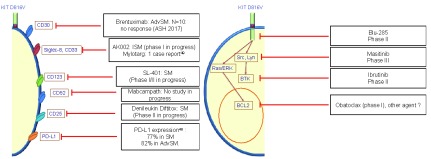
Therapeutic perspectives for mastocytosis ^48, 49^. AdvSM, advanced systemic mastocytosis; ISM, indolent systemic mastocytosis; SM, systemic mastocytosis. Based on clinicaltrials.com

The more active areas of research are targeted therapies, particularly the tyrosine kinase inhibitor avapritinib. No studies are currently investigating chemotherapies or checkpoint inhibitors. Given the overexpression of PD-L1 in SM, anti-PD1 and anti-PDL1 antibodies might be effective with an acceptable safety profile ^51^. Molecules that target extracellular antigens might be one option ( Figure 5) ^51, 52^. Given that tumor MCs from a subset of patients with AdvSM express CD30, brentuximab has been assessed ^53^. The results were very disappointing; none of the 10 patients responded to treatment ^54^. Other molecules (AK002, SL-401, and denileukin diftitox) are currently in early phase clinical development. Other treatments targeting signaling pathways are being studied ( Figure 5); avapritinib or BLU-285 (a new-generation tyrosine kinase inhibitor targeting KIT and its mutants) appears to be the most promising of these. Indeed, the results of a phase I trial (reported at the 2017–2018 American Society of Hematology meeting) highlighted a response in 13 of the 18 treated patients (72%) and, strikingly, included two complete responses. An international phase II study is in progress (the PATHFINDER study, NCT03580655), and the results are particularly expected to assess the adverse events and the long-term response. Lastly, preclinical studies for another tyrosine kinase inhibitor, DCC-2618, have reported interesting results and the drug is currently in phase I study in AdvSM (NCT02571036) ^55^. In case of confirmation of the efficacy of the two last medications mentioned, a phase III study versus midostaurin seems unlikely regarding the low prevalence of AdvSM. However, that would allow us to optimize the management of our patients according to the safety profile and the patients' comorbidities.

## Concluding remarks

Mastocytosis is a rare disease, the classification, physiopathology, and treatment of which have been greatly refined over the last decade. The discovery of *KIT* mutations has shed light on the physiopathology of mastocytosis and has provided a rationale for targeted treatment. The development of KIT-blocking tyrosine kinase inhibitors has increased overall survival rates among patients with aggressive forms of mastocytosis and has improved quality of life for patients with indolent forms of the disease. However, a better understanding of the role of additional somatic mutations should provide better prognoses, open up new treatment options, and optimize the use of ASCT (currently the only curative treatment). Many questions have yet to be answered, including the cause and physiopathology of spontaneous regression in pediatric forms, the mechanisms by which *KIT* mutations and other somatic mutations cooperate, the respective roles of the genetic background and environmental factors in disease development, and the heterogeneity of the disease’s signs and symptoms. Answers to these questions would not only provide new treatment options for mastocytosis but also help to resolve a number of problems in oncology and in allergic, neurologic, and psychiatric diseases.

## Abbreviations

AdvSM, advanced systemic mastocytosis; ASCT, allogeneic stem cell transplantation; ASM, aggressive systemic mastocytosis; BM, bone marrow; CEREMAST, French Reference Center for Mastocytosis; CM, cutaneous mastocytosis; ISM, indolent systemic mastocytosis; MC, mast cell; MCAS, mast cell activation syndrome; MCL, mast cell leukemia; MPCM, maculopapular cutaneous mastocytosis; pegIFN, pegylated interferon; SM, systemic mastocytosis; SM-AHN, systemic mastocytosis with an associated hematologic neoplasm; SSM, smoldering systemic mastocytosis; WHO, World Health Organization
